# Isolation and drug susceptibility pattern of uropathogens in Saudi diabetic and non-diabetic patients with urinary tract infection

**DOI:** 10.6026/97320630018710

**Published:** 2022-08-31

**Authors:** Ziaullah Mirza Sain, Misbahuddin Rafeeq, Hussam Aly Sayed Murad, Muhammad Barkaat Hussain

**Affiliations:** 1 Department of Microbiology, Faculty of Medicine, Rabigh campus, King Abdulaziz University, Saudi Arabia, 21589; 2 Department of Pharmacology, Faculty of Medicine, Rabigh campus, King Abdulaziz University, Saudi Arabia, 21589; 3 Department of Pharmacology, Faculty of Medicine, Ain Shams University, Cairo, Egypt

**Keywords:** Urinary tract infection, Uropathogens, Antibacterial resistance, Antibiotics, Diabetic patients

## Abstract

Urinary tract infection (UTI), contribute substantially to healthcare burden. Diabetes predispose to UTI with high glycosuria being fertile medium for bacterial growth. With changing bacterial drug resistance patterns; the problem needs to be studied periodically to ensure a rational therapy, minimize adverse effects, and cost. Therefore, it is of interest to compare the profile and susceptibility pattern of uropathogens isolated from diabetic and non-diabetic patients with UTI. Mid-stream urine samples of 1100 patients (diabetic and non-diabetic), presenting with UTI symptoms were aseptically collected and inoculated into CLED medium. Colony counts of 105cfu/ml or 104cfu/ml and >5 pus cells per high power microscopic field were regarded as significant bacteriuria. Colonies from CLED were sub-cultured onto sheep blood agar and MacConkey agar. Bacterial identification was performed on the basis of colony morphology, gram staining, and series of biochemical tests though Analytical Profile Index (API) test strips. Drug susceptibility was done by standard Kirby-Bauer disk diffusion. Data was analyzed by SPSS ver. 25.Clinically significant bacteriuria was 32.8% and 19.2% in diabetics and non-diabetics respectively. The frequency of male and female patients was 153 and 208 in diabetic group; and 69 and 142 respectively in non-diabetic group. Diabetics were twice at risk of UTI; [Odds ratio; 2.04 (CI: 1.68-2.48, p<0.05)]. .Escherichia coli and klebsiella were most common gram-negative, while Staphylococcus aureus and Coagulase-negative staphylococci (CoNS) were most common gram-positive bacteria in both the groups. Most effective antibiotics against gram-negative bacteria were carbapenems, amikacin, colistin, and piperacillin/tazobactam; while ampicillin/amoxicillin, fluoroquinolones and cephalexin were least effective. For gram-positives, vancomycin, linezolid and tigecycline were most effective. No significant difference in bacterial profile and susceptibility pattern was found between diabetics and non-diabetics. However, diabetics were twice at risk of UTI compared to non-diabetics.

## Background:

Urinary tract infection (UTI), if not treated appropriately, may lead to severe complications like acute and chronic pyelonephritis, renal scarring, loss of renal mass and function, and eventually end organ damage [1]. UTI accounted for approximately 5% of the entire annual emergency reporting by adult's ≥ 65 years in the US [2]. The situation in long-term care facilities is grimmer, where UTI comprised of approximately 30-40% of all infections [3]. In Saudi Arabia also, there is a high prevalence depending on the patient categories and associated comorbidities, with UTI being the second commonest in hospitalized patients [4]. Common bacterial isolates from UTI patients consists of Escherichia coli (E. coli) and Klebsiella pneumonia (K. pneumonia), Proteus spp., Enterococcus faecalis (E. faecalis), and Staphylococcus spp [1]. However, the spectrum varies depending on various factors like age, gender, pregnancy, past history of UTI, immunocompromised, diabetics/other chronic diseases, OPD/admitted patients etc. A study from Saudi Arabia revealed E. coli (49.1%), Klebsiella (30.7%) and E. faecalis (13.2%) respectively as the commonest pathogens in UTI, with norfloxacin, nalidixic acid and imipenem being the most effective antibiotics [5], while another study reported E. coli (37.3%), Klebsiella (16.4%), and Pseudomonas (15.7%) as the most common, with ceftriaxone, imipenem, norfloxacin, and nalidixic as having better efficacy, compared to other antibiotics [6]. Furthermore, a review about the isolate spectrum and susceptibility pattern in Saudi Arabia and other GCC nations revealed E. coli, Klebsiella, Pseudomonas, and Clostridium as the most prevalent and susceptibility was highest for imipenem (98.8%), amikacin (53.2%), gentamicin (52.3%), and ciprofloxacin (50.7%), and least for ampicillin (34.2%), and norfloxacin (40.4%). Multidrug resistance was found maximally in E. faecalis, followed by E. coli and P. aeruginosa respectively [7]. Diabetic population is especially prone to develop UTI. High sugar acts as fertile medium for bacterial growth. In addition, accompanying immune dysfunction, and dysfunctional bladder also play a role. A positive correlation was found between diabetes and increased incidence of UTI in various studies [8]. Nonetheless, there are conflicting reports about uropathogens between diabetics and non-diabetics. Some studies report similar uropathogens pattern while others report the differences between two groups [9-10]. Given the increased risk of UTI in diabetics, unequivocal bacterial pattern, frivolous usage of antimicrobials, and changing resistance patterns [5-7], the problem of UTI drug resistance needs to be studied periodically, to ensure a rational therapy, better prognosis, and minimize adverse effects and costs. Therefore, it is of interest to investigate the pathogen spectrum, and antibiotic susceptibility pattern from the urine of diabetic and non-diabetic patients presenting with complaints of UTI.

## Methods:

### Study design:

Observational, cross sectional, non-randomized, single-centre study

### Study site:

Department of Microbiology and Department of Pharmacology Faculty of Medicine in Rabigh, King Abdulaziz University, Saudi Arabia

### Study population:

After approval of study protocol, urine samples from type II diabetic patients aged ≥18 years of both genders, having any of the symptoms of UTI, were collected. For every diabetic patient, a sample from cross matched non-diabetic patient was also taken. Informed consent was waived off as the study was observational and non-interventional. Only the samples having significant bacteriuria from both the groups were included for statistical analysis.

### Exclusion criteria:

a) pregnant, b) hospitalized patients, c) having administered antibiotic within last two weeks, d) refusal to participate in the study, e) acute/chronic renal failure, f) urinary tract anomalies due to anatomical/neurological reasons, g) Immunosuppressant/corticosteroids therapy, and h) no symptoms suggestive of UTI.

### Sampling:

Clean catch midstream urine samples were collected in a wide mouth 20 mL calibrated sterile universal container. The containers were labeled, transported to the laboratory, and analyzed within one hour.

### Bacterial isolation and identification:

Isolation of bacteria was done by a calibrated loop semi-quantitative method. A calibrated loop made of sterile 4.0 mm platinum wire was used to inoculate 1µl urine on CLED, and then sub-cultured on sheep blood agar and MacConkey agar media. The plates with inoculation were incubated at 37°C for 24 h, and further extended to 48 h in case of negative results. UTI was confirmed if the concentration of the pathogenic organism cultured was ≥105 cfu/mL or 104cfu/ml and >5 pus cells per high power microscopic field [11]. The samples with positive findings underwent further processing for bacterial identification and susceptibility. Bacterial identification was done through colony morphology, gram staining, and series of biochemical tests though the Analytical Profile Index (API) test strips 20E, 20NE, 20 STREP, and API STAPH according to manufacturer's protocol (BioMerieux, France).

### Antibiotic susceptibility testing:

Antimicrobial susceptibility of isolates was done by standard Kirby-Bauer disk diffusion method [12], using commercial disks according to the guidelines of Clinical and Laboratory Standards Institute (CLSI) [13]. After obtaining pure culture, bacterial colonies were suspended and mixed gently in 5mL normal saline to make it homogenous, and turbidity adjusted to 0.5 McFarland standards. They were then inoculated evenly on Muller-Hinton agar (Oxoid) through sterile cotton swabs. The plates were left to dry at room temperature for 3-5 minutes. Sterile forceps were used to place antibiotic disks (Oxoid) on the surface and pressed gently. They were left for one hour at room temperature for optimal diffusion of antibiotics into the medium, and then further incubated at 37°C for 24 hours. Following antibiotics were selected based on regular empirical therapy by the physicians. ampicillin, amoxicillin/clavulanic acid, piperacillin/tazobactam, cephalexin, ceftazidime, cefipime, cefoperazone/sulbactam, ceftriaxone, cotrimoxazole, colistin, vancomycin, nalidixic acid, levofloxacin, norfloxacin, ofloxacin, ciprofloxacin, imipenem, meropenem, ertapenem, linezolid, nitrofurantoin, clindamycin, tigecycline, teicoplanin, amikacin, gentamycin. Sensitivity pattern was reported to the treating clinician [13].

### Statistical analysis:

SPSS ver. 25 was utilized for statistical analysis. Descriptive analysis was done and data was entered as Mean (±S.D), numbers, percentage and confidence interval. Chi-square (χ2) test was used to compare qualitative data. A value of p<0.05 was considered statistically significant.

## Results:

Out of a total of 2200 screened patients (1100 from each group), there were 609 (55.36%) males and 491 (44.64%) females in diabetic group, while non-diabetic group comprised of 472 (42.9%) males and 628 (57.1%) females as depicted in Figure 1(see PDF).

Overall, about 1/3rd of the total females, and 1/5th of the total males (from both the groups consolidated), were identified with clinically significant bacteriuria. Thus, there was a preponderance of females having UTI. In the diabetic group, the prevalence of clinically significant bacteriuria was 32.8%, while in non-diabetic group, it was 19.2%. Further analysis revealed that a total of 42.38% males and 57.62% females in diabetic group, and 32.5% males and 67.5% females in non-diabetic group were affected. Diabetic patients were having approximately twice the risk of UTI as compared to non-diabetics [Odds Ratio; 2.04, CI:1.68-2.48, p<0.05]. Maximum cases were found in patients aged≥50years in both the genders across the two groups. Mean age of males and females in diabetic and non-diabetic groups were (58.34±6.83; and 57.45±8.33), and (59.21±10.49; and 56.88±7.84) years respectively. Figure 2(see PDF) shows the age and sex distribution of the study population.

As demonstrated in Table 1 & 2(see PDF), microorganisms mainly belonging to 10 different species were isolated. Gram-negative bacteria constituted about 80.3% and 76.9% of the total isolate in diabetic and non-diabetic group respectively, while the remaining was gram-positive. Few isolates of fungi candida spp. were also reported from each group. In both groups, E. coli, followed by K. pneumoniae were most common isolates. However, it is interesting to note that the third most common gram-negative isolate was different, i.e., P. aeruginosa in diabetic group and Proteus spp. in non-diabetic group. Most common gram-positive isolates included S. aureus, followed by coagulase negative staphylococcus and E. faecalis in both the groups. Rarely other bacteria consisting of S. agalactiae S. saprophyticus were also found. Diabetic patients also revealed a greater frequency of candida infection than non-diabetics.

Antimicrobial susceptibility pattern of uropathogens isolated from diabetics and non-diabetics patients are shown in Table 1 & 2(see PDF). Overall, in diabetics, gram-negative bacteria demonstrated high sensitivity to amikacin, gentamicin, cefoperzone/sulbactam, colistin, piperacillin/tazobactam, and carbapenems; but substantial resistance to penicillin (ampicillin, amoxicillin), cephalosporins (cephalexin, ceftazidime, but not cefoperazone), nalidixic acid, and fluoroquinolones (except ofloxacin). Gram-positive isolates showed 100% susceptibility to teicoplanin, linezolid, vancomycin, and resistance to fluoroquinolones. Individually, E. coli was found to be highly susceptible to carbapenems and colistin (100%), tigecycline and amikacin (95.6% and 91.1%), gentamicin, piperacillin / tazobactam, and nitrofurantoin (86.6, 84.4, and 82.2%) respectively, and showed limited susceptibility (20-40%) to amoxicillin, ampicillin, cephalexin, ceftazidime, cotrimoxazole, nalidixic acid, and norfloxacin. Klebsiella isolates also demonstrated almost a similar pattern to E. coli, showing high resistance to cephalexin and ampicillin (93.3%). In addition, Klebsiella also depicted fair resistance to nitrofurantoin in contrast to E. coli. Furthermore, as compared to E. coli, it showed better sensitivity towards fluoroquinolones. Pseudomonas demonstrated sufficient susceptibility to carbapenems and colistin (90-100%), followed by amikacin, gentamycin and cephalosporins (70-80%), but considerable resistance to nitrofurantoin (70%). Proteus spp demonstrated high sensitivity to amikacin and carbapenems (100%) followed by third generation cephalosporins and fluoroquinolones (80%) except ciprofloxacin, while they were found to be sufficiently resistant to nalidixic acid and nitrofurantoin (80%). Citrobacter spp. showed full sensitivity to imipenem (100%), but limited sensitivity to cefipime and norfloxacin (33.3%). Acinetobacter was fully sensitive (100%) to piperacillin/tazobactam and 50% to carbapenems, ceftazidime, cefepime, amikacin and cotrimoxazole.

In gram-positive microorganism group, S. aureus depicted 100% sensitivity to teicoplanin, linezolid and vancomycin, 80% sensitivity to amikacin and clindamycin and limited sensitivity (30-40%) to fluoroquinolones. Coagulase negative staphylococcus also showed a similar pattern to S. aureus but less sensitivity to nitrofurantoin, amikacin, and amoxicillin. E. faecalis also demonstrated 100% sensitivity to teicoplanin, linezolid and vancomycin, and very limited sensitivity (33.3%) to nalidixic acid and fluoroquinolones. The pattern in non-diabetics was generally comparable with that seen in diabetic group, with sensitivity percentages slightly on a higher side for few drugs and on lower side for others. For example, sensitivity of E.coli to amoxicillin and ceftazidime was more in non-diabetics (61.1 vs 44.4%) and (50% vs 31.1%) respectively. However, sensitivity for cefoperazone/sulbactam was only 33.3% as compared to 88% in diabetic. For K.pneumoniae as well, sensitivity for piperacillin/tazobactam was high in non-diabetics (94.4% vs 73.3%), while it was less for norfloxacin (38.8%) as compared to diabetic group (53.3%). P.aeruginosa and Proteus spp. revealed similar susceptibilities to amikacin, cefepime, and ceftriaxone. Citrobacter spp. also showed comparable susceptibility to amikacin, gentamicin, levofloxacin and ertapenem. Acinetobacter also depicted slightly more susceptibility to some antibiotics, but decreased susceptibility to cotrimoxazole and ciprofloxacin (50 vs 33.3%). For gram-positive isolates, S.aureus, E.faecalis and coagulase negative staphylococcus also demonstrated parallel susceptibilities to all antibiotics in non-diabetics and diabetics.

## Discussion:

UTI is a substantial healthcare burden, and irrational antibiotic prescription has made the problem worse. Present study evaluates the spectrum and sensitivity pattern of uropathogens in diabetic and non-diabetic patients. Elderly (>50 years) patients were more affected. This is anticipated due to several factors like urine incontinence, prostatic abnormalities, and perineal muscle weakness. A report by Mahesh et al. 2010 also reinforces our findings [14]. Moreover, as anticipated, females were more affected, which can be explained by female anatomy, contraceptive use, and menopause. Diabetic patients were found to be twice at risk of UTI than non-diabetics. This is in agreement with a report by Kumar et al. 2019 [15]. Our study also revealed the prevalence of UTI as 32.8% in diabetics and 19.2% in diabetics which is slightly higher than a previous report from Saudi Arabia but in agreement with other studies from neighboring countries [16-17]. The infection is more severe and carries worse outcomes in diabetics. Several factors like growth of pathogenic bacteria due to higher glucose load in urine, impaired immune system, dysfunctional voiding due to autonomic neuropathy, and possible risk with use of SGLT2-inhibitors which increase glycosuria [18], may play key roles. It is reported that the structure of type-1 fimbriae receptors is altered in diabetics, which leads to enhanced adherence of E. coli to uroepithelial cells [19]. Also, a decrease in leukocyte chemotaxis and adhesion, and reduced concentrations of IL-6 and IL-8 predispose to UTI in diabetics [20]. Overall, the bacterial isolate pattern was more or less similar in both the groups with the exception of pseudomonas which is more common in diabetics. On the other hand, proteus was more prevalent in non-diabetic patients. Our findings agree with previous reports from Saudi Arabia and globally that E. coli followed by klebsiella and pseudomonas is the most common microorganism [5, 21-22]. However, other studies from Asian subcontinent reported a different pattern mentioning pseudomonas, citrobacter, candida spp, and some other microorganisms at the second position after E. coli [23-25]. The apparent disparity in microorganism isolate pattern can be explained partly by different study designs, for example, inclusion of admitted patients who have undergone catheterization or any surgical procedure, other comorbidities and co-infection with other pathogens. Most effective antibiotics against gram negative microorganisms in our study are carbapenems, amikacin, colistin, and piperacillin/tazobactam; while ampicillin/amoxicillin and cephalexin are least effective, which are similar to previous studies [26-27]. Fluoroquinolones and nalidixic acid were used very frequently in the past but showed high resistance in our study. Rampant and injudicious use as an empirical treatment before waiting for culture results, altered bacterial genetics, and changing virulence factors of microorganisms have played a role in their resistance [28]. Fluoroquinolones resistance studies across the globe have also reported its resistance in the range of 6-75% [29]. Nitrofurantoin has shown moderate sensitivity against E.coli but considerable resistance against other microorganisms in our study which is in agreement with a report from Saudi Arabia [30]. But in contrast, Biradar et al. 2013 reported high sensitivity of E.coli to nitrofurantoin and other common gram-negative isolates [31]. In addition, Singh et al. 2015 also advocated nitrofurantoin as an alternative, especially with increasing resistance to costly 4th generation antibiotics [32]. Furthermore, Nitrofurantoin is also considered as an alternative in treatment of VRE, and MRSA by Pulcini et al. 2012 [33]. Hence, the sensitivity pattern changes with local prescription practices, patient profile, disease spectrum and factors related to microorganisms which are discussed elsewhere. Cotrimoxazole showed considerable resistance in both the groups. A report by wright et al. 1999, mentioned an association between cotrimoxazole resistance and diabetes mellitus. However, they also included hospitalized patients [34], which is not the case in our study. For gram-positives, vancomycin, linezolid and tigecycline were most effective, but nitrofurantoin depicted moderate resistance. He et al. 2018, also reported high sensitivity of gram-positives to vancomycin, and some activity against VRE as well [27]. Most of the gram-positives were resistant to amoxicillin, fluoroquinolones, and cotrimoxazole which are in accordance with previous study from Saudi Arabia [30]. Nevertheless, diabetes predisposes to an increased risk of UTI, however, the resistance and sensitivity pattern to common antibiotics is almost similar in diabetic and non-diabetic patients. Previously Meiland et al. 2004 also observed similar pattern of E.coli resistance in both diabetic and non-diabetics [35]. Another report also concluded no significant difference in sensitivity pattern between the two groups. Hence, diabetes per se, do not affect the sensitivity pattern of microorganism against commonly prescribed antibiotics.

## Conclusions:

Identification and research into bacterial drug sensitivity patterns are warranted from time to time especially with regard to common infectious diseases in the community. UTI, especially in diabetic patients, being one of the commonest healthcare problems needs timely intervention with the right drug in the right dose at the right time to alleviate adverse effects and minimize socioeconomic burden. Our study found no significant differences in the bacterial profile and susceptibility pattern between diabetic and non-diabetic patients. However, diabetics and female patients were at higher risk for UTI.

## Limitation of the study:

As the study population was from out-patient departments, hence, regular follow-up could not be done. Admitted patients with co-morbidities might have changed the isolation and sensitivity pattern. In addition, many demographic parameters were not taken; and a relationship between isolation and sensitivity pattern, and other parameters could not be established.

## Funding:

This project was funded by the Deanship of Scientific Research (DSR) at King Abdulaziz University, Jeddah, under grant number (G-437-828-1439). The authors, therefore, acknowledge and thank DSR for the technical and financial support. DSR has no role in the study design, collection, analysis and interpretation of the data, writing of the report, or the decision to submit the article for publication.
